# MicroRNA-184 inhibits neuroblastoma cell survival through targeting the serine/threonine kinase *AKT2*

**DOI:** 10.1186/1476-4598-9-83

**Published:** 2010-04-21

**Authors:** Niamh H Foley, Isabella M Bray, Amanda Tivnan, Kenneth Bryan, Derek M Murphy, Patrick G Buckley, Jacqueline Ryan, Anne O'Meara, Maureen O'Sullivan, Raymond L Stallings

**Affiliations:** 1 Department of Cancer Genetics, Royal College of Surgeons in Ireland, York House, York Street, Dublin 2, Ireland; 2 National Children's Research Centre, Our Lady's Children's Hospital, Crumlin, Dublin 12, Ireland; 3 Department of Oncology, Our Lady's Children's Hospital, Crumlin, Dublin 12, Ireland; 4 Department of Pathology, Our Lady's Children's Hospital, Crumlin, Dublin 12, Ireland

## Abstract

**Background:**

Neuroblastoma is a paediatric cancer of the sympathetic nervous system. The single most important genetic indicator of poor clinical outcome is amplification of the *MYCN *transcription factor. One of many down-stream MYCN targets is miR-184, which is either directly or indirectly repressed by this transcription factor, possibly due to its pro-apoptotic effects when ectopically over-expressed in neuroblastoma cells. The purpose of this study was to elucidate the molecular mechanism by which miR-184 conveys pro-apoptotic effects.

**Results:**

We demonstrate that the knock-down of endogenous miR-184 has the opposite effect of ectopic up-regulation, leading to enhanced neuroblastoma cell numbers. As a mechanism of how miR-184 causes apoptosis when over-expressed, and increased cell numbers when inhibited, we demonstrate direct targeting and degradation of *AKT2*, a major downstream effector of the phosphatidylinositol 3-kinase (PI3K) pathway, one of the most potent pro-survival pathways in cancer. The pro-apoptotic effects of miR-184 ectopic over-expression in neuroblastoma cell lines is reproduced by siRNA inhibition of *AKT2*, while a positive effect on cell numbers similar to that obtained by the knock-down of endogenous miR-184 can be achieved by ectopic up-regulation of *AKT2*. Moreover, co-transfection of miR-184 with an *AKT2 *expression vector lacking the miR-184 target site in the 3'UTR rescues cells from the pro-apoptotic effects of miR-184.

**Conclusions:**

*MYCN *contributes to tumorigenesis, in part, by repressing miR-184, leading to increased levels of *AKT2*, a direct target of miR-184. Thus, two important genes with positive effects on cell growth and survival, *MYCN *and *AKT2*, can be linked into a common genetic pathway through the actions of miR-184. As an inhibitor of *AKT2*, miR-184 could be of potential benefit in miRNA mediated therapeutics of *MYCN *amplified neuroblastoma and other forms of cancer.

## Introduction

Neuroblastoma is a paediatric cancer of the sympathetic nervous system and accounts for approximately 15% of all childhood cancer related deaths. The disease has a highly varied clinical outcome, some tumours can spontaneously regress without treatment, while others can progress and lead to the death of the patient in spite of intensive multi-modal chemotherapy. Amplification of the *MYCN *transcription factor is the single most important prognostic indicator of poor patient survival and determination of genomic *MYCN *copy number status plays a major role in the stratification of patients for treatment [1]. This oncogenic transcription factor is responsible for the dysregulation of numerous genes and genetic pathways in neuroblastoma [2], and more recently it has become apparent that MYCN is also responsible for the dysregulation of microRNA [3-6].

MicroRNAs are a class of small (19-25 nt) noncoding regulatory RNAs that regulate gene expression through their binding to sites within the 3'UTR of an mRNA target gene, causing either mRNA degradation or translational inhibition [7]. These small non-coding molecules have a major role in the control of many normal cellular processes, such as cell division [8,9] or differentiation [10], and their dysregulation plays a major role in many forms of cancer [11], including neuroblastoma, as shown by expression profiling and functional studies [3-6,12-19].

Through miRNA expression profiling of different genetic subtypes of neuroblastoma, Chen and Stallings [3] and others [5,19,20] previously demonstrated that several miRNAs are differentially expressed in these tumors, particularly in regard to MYCN amplified (MNA) versus non-MNA tumor subtypes. One of the miRNAs that was expressed at lower levels in the MNA tumors relative to non-MNA tumors was miR-184, which was demonstrated to cause a decrease in cell numbers and an increase in caspase mediated apoptosis when transiently transfected into both MNA and non-MNA neuroblastoma cell lines. In this report, we identify the important molecular mechanism by which miR-184 exerts its negative effects on neuroblastoma cell survival, which involves the direct targeting of the 3'UTR of *AKT2 *mRNA, a major downstream effector of the phosphatidylinositol 3-kinase (PI3K) pathway, an important pro-survival pathway in cancer [21-23]. Thus, MYCN causes enhanced tumorgenicity, in part, through repressing a miRNA that targets this important pro-survival gene, never previously associated with neuroblastoma pathogenesis.

## Materials and methods

### Human Tissue Samples

Neuroblastoma tumour samples were obtained from patients at Our Lady's Hospital for Sick Children in Crumlin, Ireland or through the Children's Oncology Group (USA) and have been previously described in aCGH [24], mRNA [25] and miRNA [3] profiling studies.

### Cell Culture

Kelly and SK-N-AS cell lines were purchased from the European Collection of Animal Cell Cultures (Porton Down, United Kingdom). SHEP-TET21 cells were obtained from Dr. Louis Chesler with permission of Prof. Manfred Schwab [26]. Kelly cells and SHEP-TET21 cells were grown in RPMI 1640 supplemented with 10% fetal bovine serum, 2 mM Glutamine and 2 mM penicillin and streptomycin (GIBCO). SK-N-AS cells were cultured in EMEM (GIBCO) supplemented with 10% fetal bovine serum, glutamine and penicillin and streptomycin.

### Transfections

Pre-miR™ and Anti-miR™ to miR-184 and negative control 1 (a scrambled oligonucleotide) were obtained from Ambion (Austin, Texas). Short interfering (si)RNAs targeting *AKT2 *were obtained from Applied Biosystems (Foster City, CA). Three different siRNAs against *AKT2 *were chosen (s1215 sense CAACUUCUCCGUAGCAGAAtt, anti-sense UUCUGCUACGGAGAAGUUGtt, s1217 sense strand UGACUUCGACUA UCUCAAAtt and anti sense strand UUUGAGAUAGUCGAAGUCAtt) (s228853 sense strand ACAACUUCUCCGUAGCAGAtt and anti sense strand UCUGCUACGGAGAAGUUGUtt).

The Pre-miR™ and Anti-miR™ to miR-184, negative control 1 and the siRNAs to *AKT2 *were introduced into the cells by reverse transfection using the transfection agent siPORT™ *Neo*FX™(Ambion). Cell culture media was changed after 8 hours to remove the transfection reagent in an attempt to avoid toxicity which may be caused by NeoFX™. Total RNA/miRNA was extracted 24, 48 and 72 hours after transfection using RNeasy Kit/mirNeasy^© ^kit (Qiagen, UK).

### Stem-loop Reverse transcription and Real-time PCR

Reverse transcription was carried out using 50 ng of total RNA with the primer specific for miR-184 and the TaqMan microRNA reverse transcription kit (Applied biosystems). qPCR was carried out on the 7900 HT Fast Realtime System (Applied Biosystems). RNU66, a small RNA encoded in the intron of RPL5 (chr1:93,018,360-93,018,429; 1p22.1), was used for normalization in miRNA studies and RPLPO ribosomal protein was used for normalization in gene expression studies (chr12: 119,118,300-119,124; 12q24.2). A relative fold change in expression of the target miRNA/gene transcript was determined using the comparative cycle threshold method (2^-ΔΔCT^).

### Significance testing for Tumour Subtypes

The significance of miRNA differential expression over tumour sub-types was evaluated by assigning P-values based on the non-parametric Mann-Whitney test.

### Cloning the Precusor miRNA-184

The stem loop precursor sequence of miR184 was cloned into the pcDNA6.2-GW/EmGFP expression vector (BLOCK-iT Pol II miR RNAi Expression Vector kit, Invitrogen). The following oligonucleotides were designed which encode the sense and antisense strands of the pre-miR184 sequence. These oligonucleotides include the appropriate 5' and 3' overhangs to facilitate cloning into the linearised pcDNA6.2-GW/EmGFP vector (supplied within the BLOCK-iT kit, Invitrogen).

Pre-miR184 Sense strand:

**TGCTG**CCAGTCACGTCCCCTTATCACTTTTCCAGCCAGCTTTGTGACTGTAAGTGTTGGCAGGAGAACTGATAAGGGTAGGTGATTGA

Pre-miR184 Antisense strand:

**CCTG**TCAATCACCTACCCTTATCAGTTCTCCTGCCAACACTTACAGTCACAAAGCTGGCTGGAAAAGTGATAAGGGGACGTGACTGG**C**

The pcDNA6.2-GW/EmGFP-miRNA-184 construct or the control construct (pcDNA6.2-GW/EmGFP-miRnegative control, Invitrogen) was transfected into Kelly and SK-N-AS cells using lipofectamine 2000 (Invitrogen, Carslbad) according to manufacturers instructions. Quantitative real-time PCR and fluorescent microscopy were carried out to determine efficient transfection and transcription of the vector.

### *AKT2 *Expression Vector

The expression vector pcDNA 3 containing *AKT2 *was obtained from Prof. Joe Testa (Fox Chase Cancer Centre, Philadelphia) [27]. 1 μg of the vector or the control empty vector was transfected into Kelly and SK-N-AS cells using Lipofectamine 2000.

### Apoptosis Assays

Apoptosis was demonstrated by annexin-V staining and propidium iodide (PI) exclusion using the FITC Annexin-V Apoptosis Detection Kit I (BD Pharmingen, San Diego, CA, USA). Briefly, adherent and supernatant Kelly cells were collected, washed twice in cold PBS, and resuspended in 1× Annexin-V binding buffer at a concentration of 1 × 10^6 ^cells/ml. An aliquot of 100 μl of suspension (1 × 10^5 ^cells) was stained with 5 μl Annexin-V-FITC and 5 μl PI, and incubated for 15 minutes at room temperature in the dark. Binding buffer (400 ul) was added and cells acquired (10,000 cells) immediately using a BD LSR II flow cytometer (Becton Dickinson, San Jose, CA, USA) and analysed using BD FACSDiva 4.0 Software. Experiments were performed in multiples to qualify apoptosis by phosphatidylserine (PS) externalization.

Cell Death was also evaluated using the 3/7 Caspase detection kit from Promega (Madison, WI). Neuroblastoma experimental cells were plated in quadruplicate in 96-well plates. 72 hours after transfection, 10 ul of caspase 3/7 was added to each well. Samples were read after 1 hr of incubation with the caspase substrate on a Viktor Microplate luminometer (Molecular Devices, Sunnyvale, CA).

### Growth Curve

For cell number assays, cells were set up in triplicate in 6 well plates. Cells were seeded at equal densities of 3 × 10^4 ^cells per well. When carrying out transfections using the microRNA mimics or anti-miRs (as described above) each time point was set up with a non-transfected (with transfection reagent) and a scrambled oligonucleotide control (negative 1). Cells were trypsinised from 6 well plates at 24, 48 and 72 hour time points, and re-suspended in 1 ml of media. A haemocytometer was used to count cell numbers. Counts from triplicate wells were averaged.

### Western Blot

Total protein was isolated from cells using a radioimmunoprecipitation assay (RIPA) lysis buffer (Sigma). Protein concentration was measured using the BCA assay from Millipore. Proteins were fractionated on 10% polyacrylamide gels, and blotted onto nitrocellulose membrane. The membrane was probed with the Anti-AKT2 Antibody (Millipore) or anti-MYCN (Abcam), anti β-Actin from (Abcam) or anti GAPDH (abcam) (used for loading controls). Signal was detected using Immoblion Western (Millipore).

### Luciferase Reporter

A 76nt long region of the 3'UTR of AKT2 containing the predicted miR-184 binding site (underlined) was ligated into the pMiR-Reporter vector (Ambion) 3' of the luciferase gene:5'CTAGTCCTCTGTGTGCGATGTTGTTATC**TGACAGTTCTCCGTCC**CTACTGGCCTTTCTCCTCGTCTTC*GCTCAGC*A 3'

As a negative control, three mutations (lower case) were introduced into the seed region of miR-184 binding site of this sequence:

5'CTAGTCCTCTGTGTGCGATGTTGTTATC**TGACAGTTCTtCaaCC**CTACTGGCCTTTCTCCTCGTCTTC*GCTCAGC*A 3'

KELLY cells were plated at 8 × 10^4 ^in 12 well format. After 24 hrs the pMir-Reporter containing the *AKT2 *binding site for miR-184 or the mutated *AKT2 *binding site were co-transfected with either the pre-miR-184 mimic or a scrambled negative control sequence using Lipofectamie 2000. All experiments were also co-transfected with the pmiR-Report β-galactosidase vector as a control for transfection efficiency and normalization. Luciferase activity was measured by One-Glo luciferase assay (Promega) according to manufactures instructions after 24 hours on the Viktor Plate Reader.

## Results

### MiR-184 expression is inversely related to *MYCN *levels

In order to experimentally determine that MYCN levels influence quantities of miR-184, we examined miR-184 expression in the SH-EP TET21 neuroblastoma cell line containing a *MYCN *construct which is repressible by doxycycline. As demonstrated by qPCR and Western blotting, addition of doxycycline to the cell culture caused a dramatic reduction in both *MYCN *mRNA and protein levels (Additional File 1a and 1b). MYCN depleted SH-EP cells had an 8 fold increase in MiR-184 levels (Additional File 1c), indicating that MYCN either directly or indirectly suppresses miR-184 transcription, consistent with our earlier expression profiling studies of primary tumors [3].

### Biological effects of MiR-184 ectopic up-regulation and endogenous down-regulation

**Figure 1:**
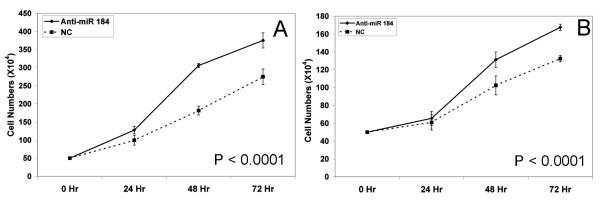
**Growth curves for Kelly (A) and SK-N-AS (B) following transfection with the anti-miR-184 (×3 biological replicates)**. A scrambled anti-miR was used as negative control.

It was previously reported that transfection of miR-184 mimics into neuroblastoma cell lines causes a decrease in cell numbers and increase in apoptosis in neuroblastoma cell lines [3]. Here, we first assessed whether the reciprocal experiment of knocking down endogenous miR-184 in Kelly and SK-N-AS cell lines following transfection with an anti-miR-184 also has a detectable biological effect. As illustrated in Figure 1a and 1b, the inhibition of miR-184 results in a reproducible and statistically significant increase in cell numbers (~1.6 fold in Kelly; p < 0.0001; and 1.3 fold in SK-N-AS; p < 0.0001) (Figure 1a and 1b). In addition, we also determined that ectopic over-expression of miR-184 at physiological levels using an expression plasmid has similar biological effects on Kelly and SK-N-AS cells as the mature miR-184 mimics which were introduced at supra-physiological levels (Additional File 2a-g). The molecular mechanism by which miR-184 exerts effects on cell numbers and apoptosis of neuroblastoma cells, however, is unknown.

### MiR-184 targets the *AKT2 *mRNA 3' UTR

**Figure 2:**
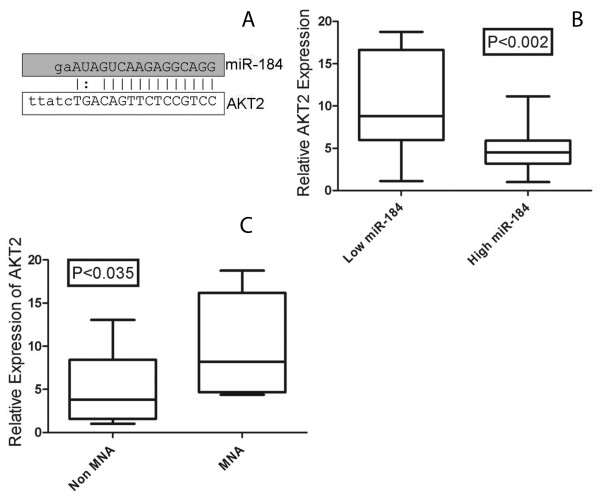
**(A) Predicted alignment of miR-184 to the mRNA 3'UTR region of *AKT2*, as predicted by the Sanger miR Registry**. (B) *AKT2 *mRNA levels were significantly different (p < 0.002) between tumours with high miR-184 levels (predominantly MNA tumors)(n = 10) and tumours with low miR-184 levels (predominantly non-MNA)(n = 9). *AKT2 *mRNA levels were also significantly different (p < 0.035) between tumours with MNA (n = 10) versus non-MNA (n = 9).

**Figure 3:**
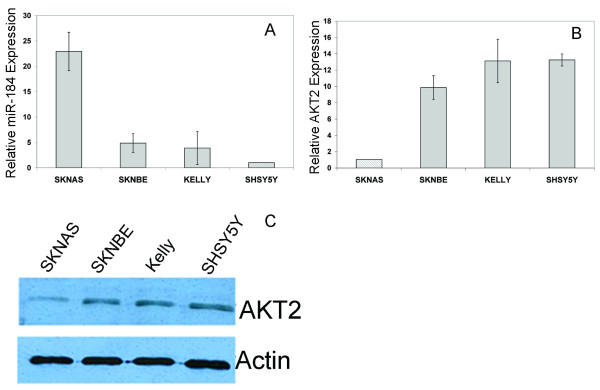
**(A) RT-qPCR analysis of miR-184 in four neuroblastoma cell lines**. RNU66 was used as an endogenous control and expression levels are relative to SH-SY5Y, set as 1.0. AKT2 mRNA (B) and protein (C) levels in the same four neuroblastoma cell lines (SK-N-AS, SK-N-BE, Kelly, SH-SY5Y). Actin was used as an endogenous loading control for the western blot, while RPLPO was used as endogenous control for RT-qPCR, all values are relative to SK-N-AS. Note that *AKT2 *mRNA levels show an inverse relationship to the endogenous miR-184 levels.

An examination of the Sanger microcosm database http://microrna.sanger.ac.uk/sequences/ indicated that miR-184 has a very large number of computationally predicted mRNA targets. Among the top 3% (n = 30) of miR-184 predicted targets was the 3'UTR of *AKT2*, which had a high level of sequence identity with the miR-184 seed region, a 13 base pair match (Figure 2a). We focused our studies on *AKT2 *as a potential miR-184 target given that this was the only gene in the top 3% whose function might account for the apoptotic phenotype induced by miR-184. *AKT2 *is a well documented pro-survival proteinFor an initial assessment of whether *AKT2 *mRNA levels and miR-184 levels might be inversely related, qPCR analysis of *AKT2 *mRNA was carried out on 10 tumors with low miR-184 and 10 with high levels. As illustrated in Figure 2b, *AKT2 *mRNA levels are significantly lower in tumors with higher miR-184 (P < 0.002). As one might expect, *AKT2 *was expressed at higher levels in MNA tumors relative to non-MNA tumors (P < 0.035), as MYCN suppresses miR-184 transcript quantities (Figure 2c). An inverse relationship between miR-184 and *AKT2 *mRNA levels was also determined to exist in neuroblastoma cell lines (Figure 3a and 3b). Levels of AKT2 protein corresponded to the levels of mRNA in the cell lines (Figure 3c).

**Figure 4:**
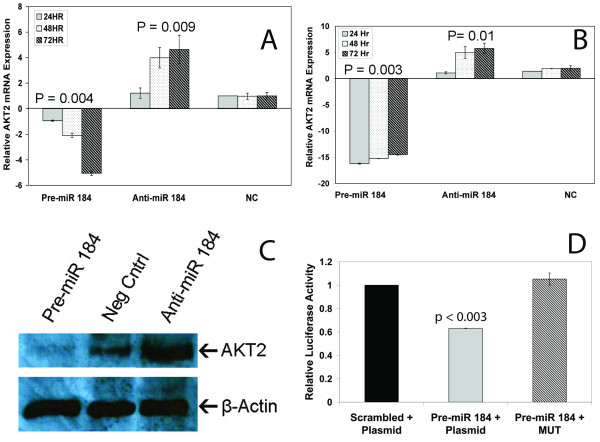
**Relative AKT2 mRNA levels following transfection of miR-184 mimics (pre-miR-184), anti-miR-184, or a scrambled oligo (NC) into (A) Kelly and (B) SK-N-AS cells at 24, 48 and 72 hrs after transfection**. RT-qPCR results were normalised to RPLPO ribosomal protein and are relative to the negative control at 24 hrs. Western Blot (C) representing protein levels of AKT2 in Kelly cells 72 hrs after transfection with the miR-184 mimics, a negative control oligo, or anti-miR-184. β-Actin was used for the endogenous control. (D) pMir-Reporter containing the *AKT2 *binding site for miR-184 in the 3' UTR region of the luciferase gene co-transfected into Kelly cells with a scrambled negative control oligonucleotide (left) or mature miR-184 mimic (middle). The right bar shows the result of co-transfection with the pMir-Reporter containing the mutated *AKT2 *binding site for miR-184 co-transfected into Kelly cells with mature miR-184 mimic. Luciferase values were normalized to B-galactosidase on a second plasmid that was co-transfected into each cell line and all values are relative to the negative control (left).

To confirm that *AKT2 *was indeed regulated by miR-184 in neuroblastoma, the miR-184 mimic and the anti-miR-184 were individually transfected into Kelly and SK-N-AS cell lines. A significant decrease of *AKT2 *mRNA was observed over three time points (24, 48 and 72 hrs) following transfection of miR-184 mimics into Kelly (P = 0.004)(Figure 4a) and SK-N-AS (P = 0.003)(Figure 4b), and conversely, the suppression of miR-184 using the anti-mir-184 caused an increase in mRNA for *AKT2 *across all time points in both cell lines (P = 0.009 and 0.01 respectively). These results were also observed at protein level (Figure 4c). MiR-184 is not predicted to target the 3'UTRs of related AKT family members, *AKT1 *nor *AKT3*. Consistent with expectations, *AKT1 *mRNA levels remained constant when miR-184 was transfected into Kelly and SK-N-AS (Additional File 3a), while *AKT3 *mRNA was undetectable in both cell lines by TaqMan qPCR.

The effect of miR-184 on AKT2 levels appears to be a direct effect of miR-184 targeting *AKT2 *mRNA, since co-transfection of a pMir-Reporter containing the *AKT2 *binding site for miR-184 and mature miR-184 mimics significantly (p < 0.003) diminished luciferase activity while co-transfection of the reporter with a negative control oligonucleotide had no effect (Figure 4d). A three base pair mutation introduced into the seed region of the miR-184 binding site in the *AKT2 *3' UTR completely abolished the ability of mature miR-184 mimics to affect luciferase activity.

### The phenotypic effects of miR-184 can be attributed to targeting AKT2

**Figure 5:**
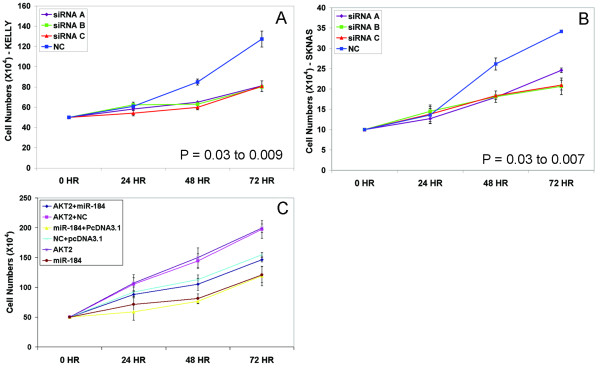
**(A) Growth curves following transfection with each *AKT2 *siRNA and negative control for (A) Kelly (p values for each siRNA ranged from 0.01 to 0.03) and (B) SK-N-AS (p values for each siRNA ranged from 0.007 to 0.03)**. Growth curves (C) for Kelly cells following transfection with various combinations of *AKT2 *expression vector, empty vector, miR-184 mimics or negative control (NC) oligonucleotides. The highest number of cells was produced by either the *AKT2 *expression vector alone or in combination with a negative control oligonucleotide. These cells had both endogenous and ectopic *AKT2 *(Additional File 5). Co-transfection with the *AKT2 *expression plasmid (lacking the miR-184 target site) and miR-184 mimics yielded a cell number curve that was not statistically different from co-transfection with a negative control oligonucleotide and the empty expression vector. The former cell population had ectopic but not endogenous *AKT2 *activity, while the latter cell population had endogenous, but no ectopic *AKT2*. Both of these cell number curves were significantly different from the other curves (p < 0.003). Transfection of miR-184 mimics by themselves or in combination with the empty vector significantly impacted cell growth, as these cells had no ectopic *AKT2 *and endogenous *AKT2 *was inhibited.

**Figure 6:**
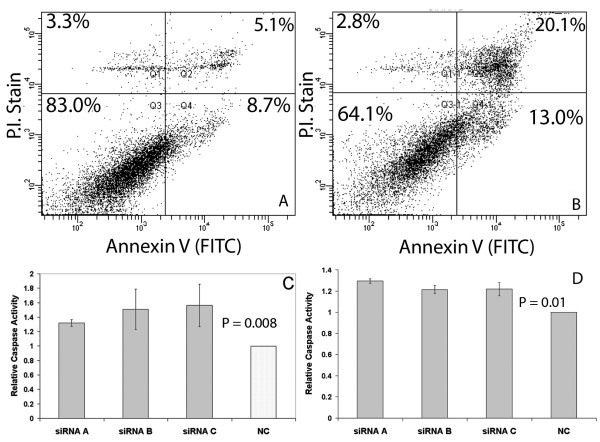
**FACs analysis of annexin V staining in Kelly cells**. (A) transfection with negative control oligonucleotide and (B) siRNA mediated knock down of *AKT2 *using siRNA. Cells were harvested for detection of externalised annexin V (apoptotic cells) and PI staining of DNA (necrotic cells). Double-stained cells (upper right quadrant) indicate cells in late apoptosis. The resultant increase in apoptosis caused by siRNA inhibition of *AKT2 *was highly significant (P < 0.0001). Analysis of caspase 3/7 activity in Kelly (C) and SK-N-AS (D) cells following siRNA knockdown of *AKT2 *relative to negative control (NC).

MiR-184 has many computationally predicted targets, so in order to determine if the anti-proliferative effects of miR-184 could be attributable to targeting *AKT2*, we transfected both Kelly and SK-N-AS neuroblastoma cell lines with three different siRNAs to *AKT2 *and examined the effects on the rate of accumulation of cell numbers. *AKT2 *mRNA knockdown ranged from 68 to 98% by 48 hrs, depending on the siRNA (Additional File 4a and 4c), with AKT2 protein being proportionally reduced (Additional File 4b and 4d). Both cell lines exhibited a marked decline in cell numbers for each siRNA relative to the negative control (Figure 5a and 5b), along with a highly significant increase (3.9 fold; P < 0.0001) in the late apoptotic cell fraction assessed by FACs analysis of an annexin V staining assay (Figure 6a and 6b). A statistically significant increase in caspase 3/7 activation in both Kelly and SK-N-AS cells following siRNA mediated *AKT2 *knock down also occurred (Figure 6c and 6d). Consistent with expectations, *AKT1 *mRNA levels remained constant when *AKT2 *siRNA was transfected into the cell lines (Additional File 3b).

To demonstrate that the increase in cell numbers that occurred following miR-184 knockdown resulted specifically from *AKT2 *up-regulation, we transfected the pcDNA3-AKT2 plasmid into Kelly neuroblastoma cells. This resulted in a 5 to 22 fold increase in *AKT2 *mRNA levels, and a 30% increase in cell numbers by the 72 hr time point relative to the negative control (p = 0.006). (Figure 5c). Since the pcDNA3-AKT2 construct lacks the miR-184 binding site in the 3' UTR, we also co-transfected pcDNA3-AKT2 along with the miR-184 mimics to determine if ectopic up-regulation of AKT2 could rescue Kelly cells from the anti-proliferative effects of miR-184. As illustrated in Figure 5c, the numbers of cells accumulated over 72 hours for Kelly cells co-transfected with pcDNA3-AKT2 and miR-184 was not statistically different to that of Kelly cells transfected with a negative control oligonucleotide and the pcDNA3 empty vector. However, this co-transfection with pcDNA3-AKT2 and miR-184 mimics yielded a cell accumulation rate that was significantly higher then cells transfected with miR-184 mimics (p <0.003) or miR-184 mimics and pc-DNA3.1 empty vector (p <0.003), indicating that ectopic *AKT2 *lacking a miR-184 binding site can rescue the cells from ectopic miR-184 up-regulation (Figure 5c). As illustrated in Additional File 5, RT-qPCR analysis of *AKT2 *mRNA indicated that there were statistically significant (p < 0.01) differences in *AKT2 *mRNA levels in each of the transfected cell populations at each time point, consistent with expectations. From all of the above experiments, we conclude that the phenotypic effects of miR-184, at least to a large extent, can be attributed to the targeting and reduction of *AKT2*.

## Discussion

This study identifies *AKT2 *as an important pro-survival gene in neuroblastoma and our results further demonstrate that *MYCN *indirectly regulates *AKT2 *through miR-184. It is unknown whether MYCN directly or indirectly suppresses miR-184 expression. There are two DNA sequence motifs, *GGCATG *and *CCCGTG*, reported to bind to MYCN at the *MCM4 *and *MCM5 *loci [28], approximately 2.6 Kb upstream of the predicted miR-184 start site, so it is possible that the suppression of miR-184 is a direct effect of MYCN binding. Examination of our MYCN chromatin immunoprecipitation data, as detailed in Murphy et al [29], indicates that MYCN binds weakly to this site, but whether this binding actually has a regulatory effect requires further experimental studies. Regardless of whether the effect of MYCN on miR-184 transcript levels is direct or indirect, we conclude that MYCN provides a tumourigenic effect, in part, by protecting *AKT2 *mRNA from degradation by miR-184, permitting this important pathway to remain functional.

Although miR-184 is predicted to target several hundred genes, several lines of evidence indicate that the targeting of *AKT2 *mRNA by itself can fully account for the observed apoptotic phenotype. First, siRNA mediated inhibition of *AKT2 *in Kelly and SK-N-AS cells induces a level of apoptosis that is comparable to miR-184 ectopic up-regulation. Second, ectopic up-regulation of *AKT2 *causes an increase in cell numbers similar to that observed following miR-184 knock-down, and the effects of ectopic miR-184 up-regulation are abrogated by ectopic over-expression of an *AKT2 *expression plasmid lacking the miR-184 binding site. We can not rule out the possibility that the targeting of other genes by miR-184 has altered the phenotypes of these cells in some undetectable manner, only that miR-184 targeting of *AKT2 *fully accounts for the pro-apoptotic effects.

*AKT2 *is a homolog of the v-akt oncogene, a protein serine/threonine kinase pro-survival protein, which is member of the AKT family of proteins (AKT1, 2 and 3) that are activated by the phosphatidylinositol 3' kinase pathway [22]. The phosphatidylinositol 3' kinase (PI3K) pathway is one of the most potent pro-survival pathways in cancer [21]. Activation of the AKT pathway through phosphorylation of serine or threonine is associated with poor clinical outcome in neuroblastoma, as demonstrated through immunohistochemical staining of tissue arrays with an antibody that co-recognizes all three AKT family members [30]. In addition, inhibition of AKT activation can prevent BDNF mediated protection of neuroblastoma cells from chemotherapy induced apoptosis [31]. Our results indicate that the *AKT2 *isoform expression levels are critical for neuroblastoma cell survival even in the absence of chemotherapeutic compounds. The other isoform which is expressed at high levels in neuroblastoma cell lines, *AKT1*, does not possess a miR-184 target site, remains constant in all of our experiments, and does not rescue the cells from the effects of miR-184 over-expression. This is consistent with findings that AKT2 does not share complementary functions with AKT1 regarding cell invasiveness and survival in other forms of cancer [32,33].

The deregulation of the AKT signalling pathway has been associated with numerous other cancers including glioblastoma, breast, prostate and lung [21]. The activation of this pathway has been associated with a more aggressive phenotype, resistance to treatment [34], and poor outcome in a large number of cancers [21]. There is still little known about the specific role of each of the three AKT isoforms, however, consistent with our result in neuroblastoma, AKT2 is emerging as one of the more important isoforms with respect to cancer. Over-expression of *AKT2 *kinase is frequently observed in ovarian cancer [35], breast cancer [36], and approximately 32% of pancreatic tumours [37]. In addition, AKT2 down-regulation sensitised ovarian cancer cells to paclitaxel induced apoptosis and indicated that AKT2 may have a more important role in drug resistance than other members of the AKT family [38]. AKT2 was also shown to reduce sensitivity to the chemotherapeutic agent, cisplatin, by regulating XIAP, an inhibitor of execution of caspase 3 [39]. Moro et al (2009) et al recently demonstrated that AKT2 and not AKT1 or AKT3 is activated in prostate cancer cells in response to oxidative stress, resulting in enhanced cell migration and cell survival. Finally, AKT2 also has been reported to be directly implicated in cell migration and invasiveness of glioblastoma [40].

There is presently not very much known about miR-184 involvement in cancer. It was reported to be up-regulated in squamous cell carcinoma (SCC) of the tongue, and suppression of this miRNA in SCC cell lines showed reduced cell numbers and an increase in apoptosis, suggesting an anti-apoptotic role for mir-184 [41]. However, this result seems contradictory to another paper published by Yu et al (2008) where miR-184 appears to have a tumor suppressive effect in SCC cell lines. Yu et al (2008) showed that miR-205 targets *SHIP2*, a protein that causes a reduction in activated phosphorylated AKT, but not in total AKT amounts. Thus, miR-205, which is elevated in aggressive SCC, is acting oncogenically by targeting *SHIP2*, allowing AKT activation. They further report that miR-184 antagonizes miR-205, so in this sense, miR-184 is acting as a tumor suppressor. The effects of ectopic miR-184 over-expression on *AKT *mRNA or protein levels was not examined by Yu et al (2008), and this was the first report of a microRNA interfering with the action of another miRNA. Our results indicating that miR-184 acts in a tumor suppressive manner in neuroblastoma does not shed further light upon these seemingly contradictory reports, as the role of any miRNA in cancer is likely to be cell context dependent.

Finally, a number of studies have sought to identify small molecule inhibitors of AKT family members for cancer therapy [42,43]. MiR-184, which targets the *AKT2 *mRNA, is a naturally occurring inhibitor of this protein, and has potential value in miRNA mediated therapeutics for any form of cancer dependent on AKT2.

## Competing interests

The authors declare that they have no competing interests.

## Authors' contributions

NHF, IB, AT, DMM, PGB, JR carried out the experimental work, KB provided data analysis, AOM and MOS provided tumor samples, clinical information, and/or histopathological analysis, NHF, IB, JR, PGB and RLS designed the study, interpreted the findings and participated in writing the paper. All authors read and approved the manuscript.

## Supplementary Material

Additional file 1 (A) Western blot showing MYCN protein in Kelly (*MYCN *amplified), SH-EP TET21 cells untreated and treated with doxycycline. GAPDH was used as the endogenous loading control. (B) Relative *MYCN *mRNA levels in SHEP TET21 cells treated (MYCN off) and untreated (MYCN on) with doxycycline as assessed by TaqMan qPCR. (C) RT-qPCR analysis of miR-184 levels in SH-EP-TET21 cells treated (MYCN off) and untreated with doxycycline (MYCN on). miR-184 expression is relative to untreated SH-EP cells. Click here for file

Additional file 2 miR-184 levels as analyzed by qRT-PCR in Kelly (A) and SK-N-AS (B) cells at 24 and 48 hours following transfection with pcDNA6.2-184 and empty vector (negative control). All values are relative to the negative control at 24 hrs, set as 1.0. Growth curves for Kelly cells (C) and SK-N-AS cells (D) after transfection with the stem loop precursor sequence of miR184 cloned into pcDNA6.2-GW/EmGFP. PcDNA6.2-GW/EmGFP-miRnegative control was used as a negative control (NC). Caspase 3/7 Assay for Kelly cells (E) and SK-N-AS cells (F) after transfection with the same stem loop precursor sequence of miR184 or the pcDNA6.2-GW/EmGFP-miRnegative control, set as 1.0. FACs analysis of annexin V staining in Kelly cells transfected with pcDNA6.2-GW/EmGFP-miRnegative control (G) and pcDNA6.2-184 (H). Click here for file

Additional file 3 (A) Relative *AKT1 *mRNA levels following transfection of Kelly cells with miR-184 mimics or negative control oligonucleotide at different time points. (B) Relative *AKT1 *mRNA levels following transfection of Kelly cells with three different *AKT2 *siRNAs or negative control siRNA at different time points. *RPLPO *was used as endogenous control for RT-qPCR and all values are relative to the negative control at 24 hrs. Click here for file

Additional file 4 Assessment of siRNA knockdown of *AKT2 *mRNA (A, C) and protein (B, D) in Kelly cells and SK-N-AS cells, respectively, by qRT-PCR or Western blot. *RPLPO *was used as endogenous control for RT-qPCR and all values are relative to the negative control at 24 hrs. Click here for file

Additional file 5 qRT-PCR assessment of *AKT2 *mRNA levels at 24, 48 and 72 hours following transfection with different combinations of plasmids and oligonucleotides. In the *AKT2 *rescue experiment (*AKT2 *plasmid + miR-184 mimics), *AKT2 *levels are not significantly different from the negative controls (pDNA3.1 empty vector + negative control oligo or negative control oligo alone) and are intermediate between cells transfected with *AKT2 *alone (cells having endogenous and ectopic AKT2) and cells transfected with miR-184 mimics alone (inhibited endogenous and no ectopic). All *AKT2 *mRNA levels are relative to the co-transfection with pcDNA3.1 empty vector and negative control at the 24 hr time point. Click here for file
