# Supplementary figures and images for: MicroRNA-184 inhibits neuroblastoma cell survival through targeting the serine/threonine kinase AKT2
Source: Mol Cancer. 2010 Apr 21;9:83. doi: 10.1186/1476-4598-9-83 (PMC2864218; doi:10.1186/1476-4598-9-83)

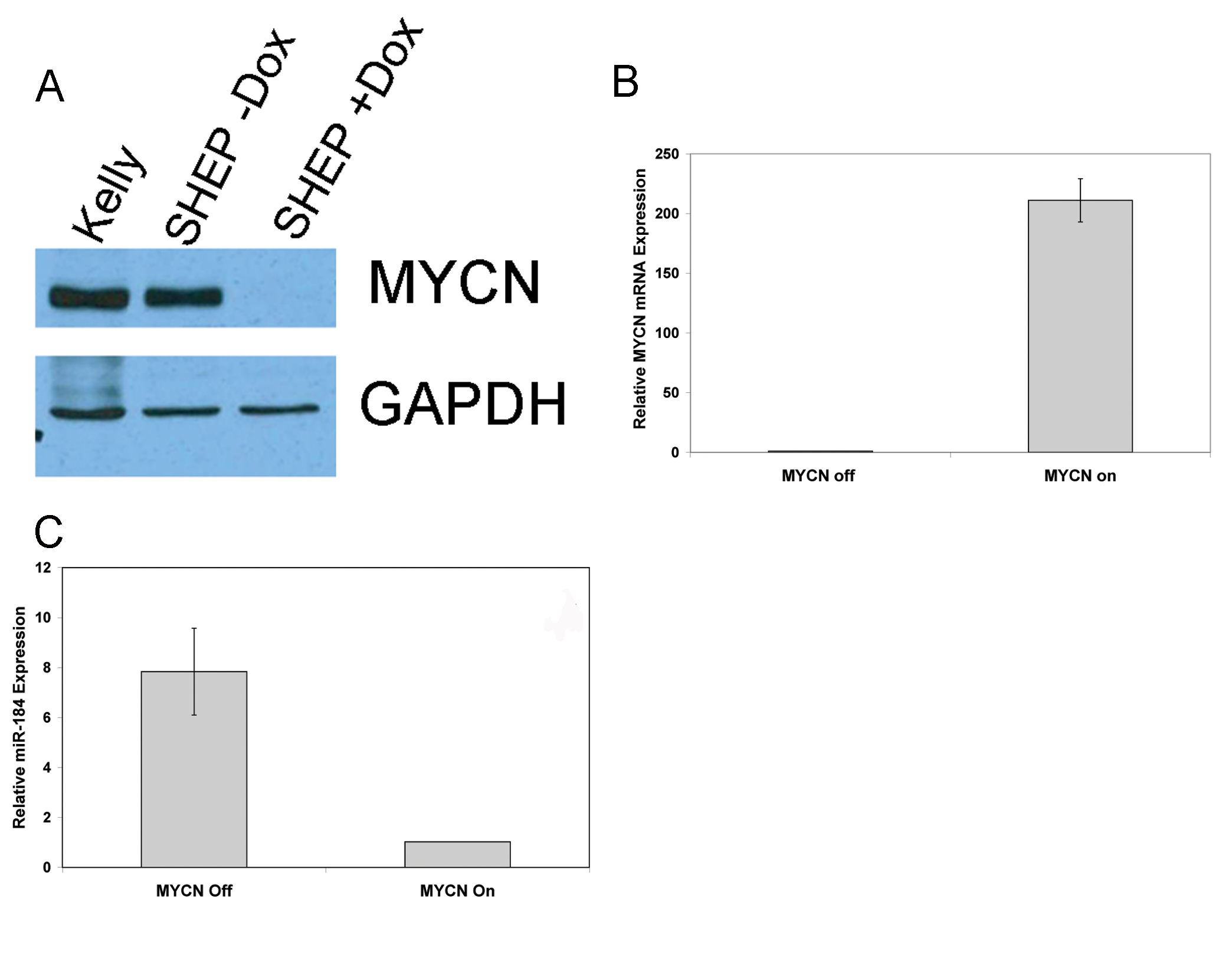

Supplement: Additional file 1 — (A) Western blot showing MYCN protein in Kelly (MYCN amplified), SH-EP TET21 cells untreated and treated with doxycycline. GAPDH was used as the endogenous loading control. (B) Relative MYCN mRNA levels in SHEP TET21 cells treated (MYCN off) and untreated (MYCN on) with doxycycline as assessed by TaqMan qPCR. (C) RT-qPCR analysis of miR-184 levels in SH-EP-TET21 cells treated (MYCN off) and untreated with doxycycline (MYCN on). miR-184 expression is relative to untreated SH-EP cells. [file 1476-4598-9-83-S1.TIFF]

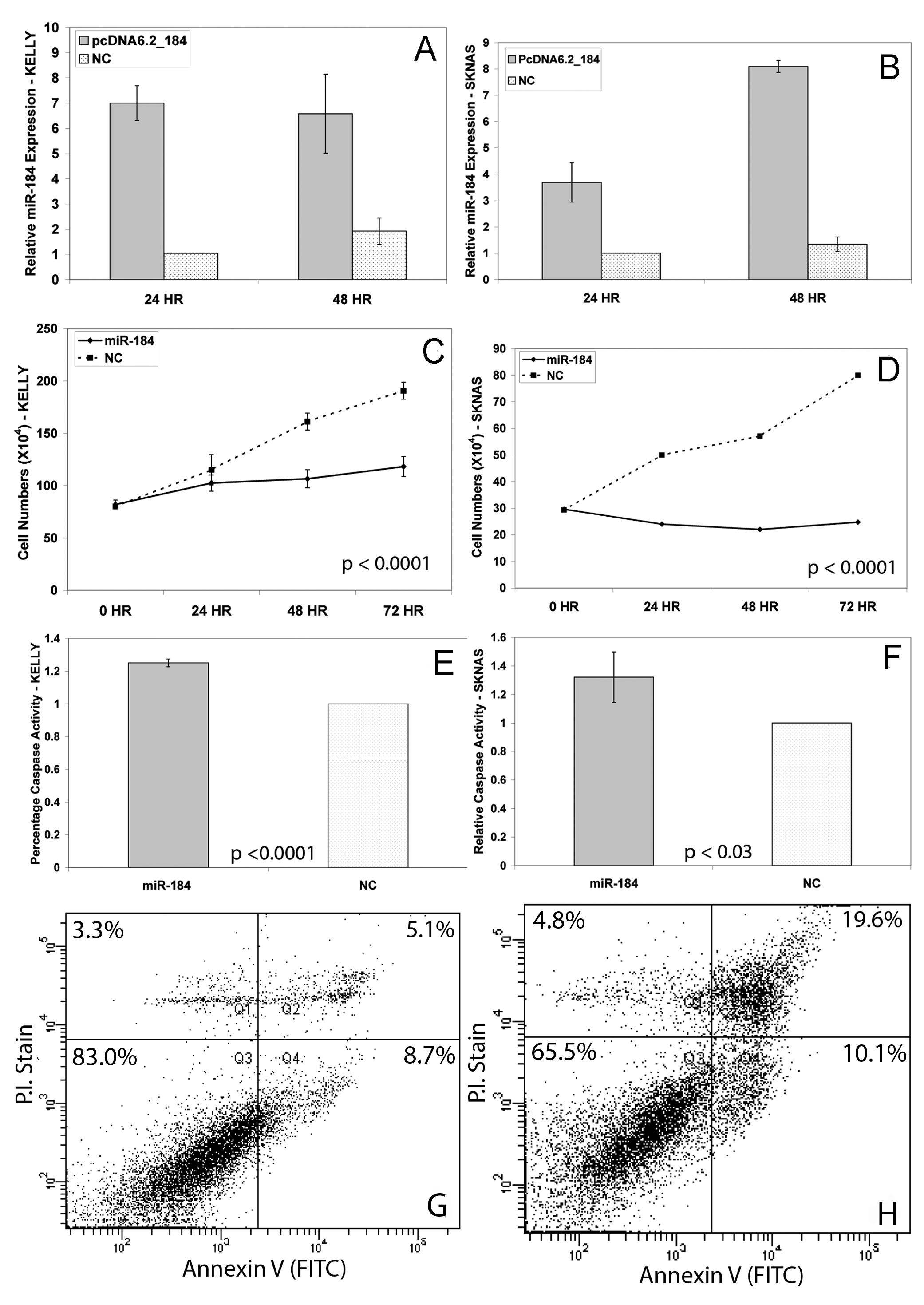

Supplement: Additional file 2 — miR-184 levels as analyzed by qRT-PCR in Kelly (A) and SK-N-AS (B) cells at 24 and 48 hours following transfection with pcDNA6.2-184 and empty vector (negative control). All values are relative to the negative control at 24 hrs, set as 1.0. Growth curves for Kelly cells (C) and SK-N-AS cells (D) after transfection with the stem loop precursor sequence of miR184 cloned into pcDNA6.2-GW/EmGFP. PcDNA6.2-GW/EmGFP-miRnegative control was used as a negative control (NC). Caspase 3/7 Assay for Kelly cells (E) and SK-N-AS cells (F) after transfection with the same stem loop precursor sequence of miR184 or the pcDNA6.2-GW/EmGFP-miRnegative control, set as 1.0. FACs analysis of annexin V staining in Kelly cells transfected with pcDNA6.2-GW/EmGFP-miRnegative control (G) and pcDNA6.2-184 (H). [file 1476-4598-9-83-S2.TIFF]

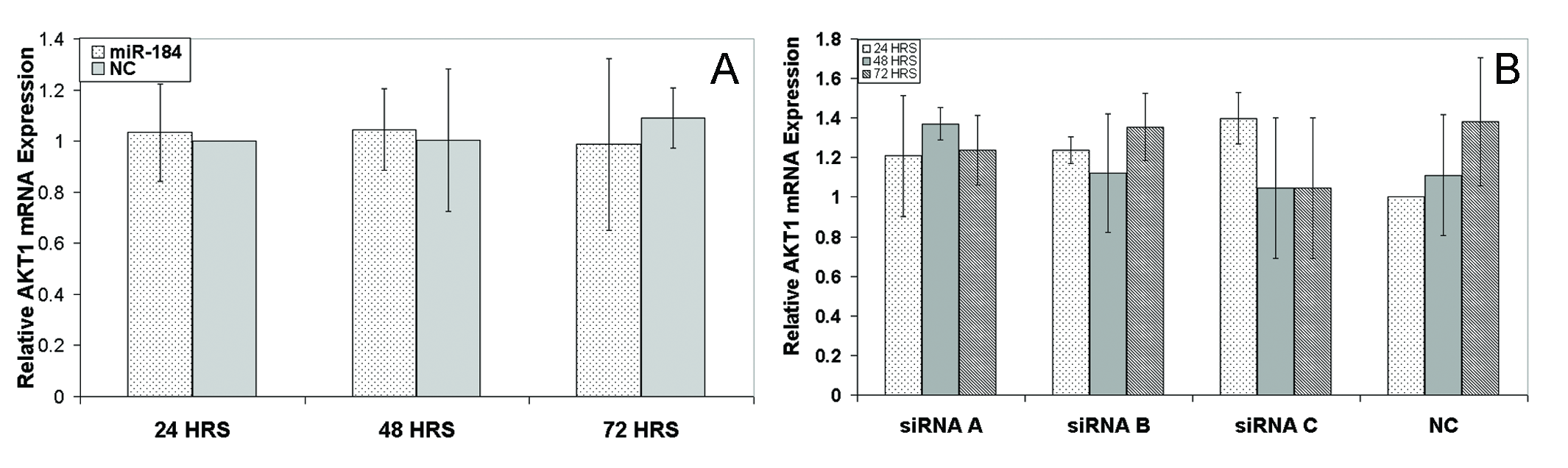

Supplement: Additional file 3 — (A) Relative AKT1 mRNA levels following transfection of Kelly cells with miR-184 mimics or negative control oligonucleotide at different time points. (B) Relative AKT1 mRNA levels following transfection of Kelly cells with three different AKT2 siRNAs or negative control siRNA at different time points. RPLPO was used as endogenous control for RT-qPCR and all values are relative to the negative control at 24 hrs. [file 1476-4598-9-83-S3.TIFF]

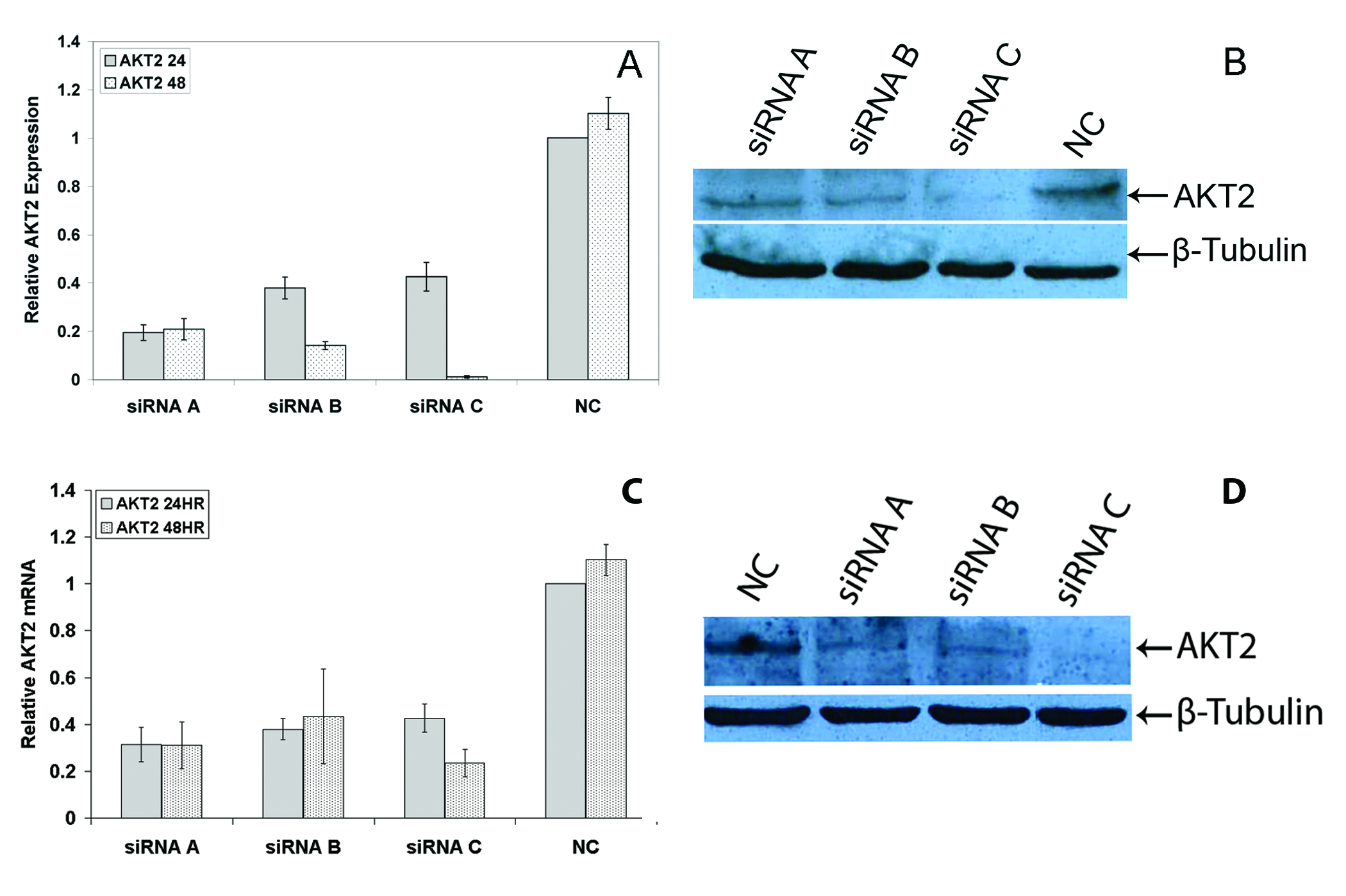

Supplement: Additional file 4 — Assessment of siRNA knockdown of AKT2 mRNA (A, C) and protein (B, D) in Kelly cells and SK-N-AS cells, respectively, by qRT-PCR or Western blot. RPLPO was used as endogenous control for RT-qPCR and all values are relative to the negative control at 24 hrs. [file 1476-4598-9-83-S4.TIFF]

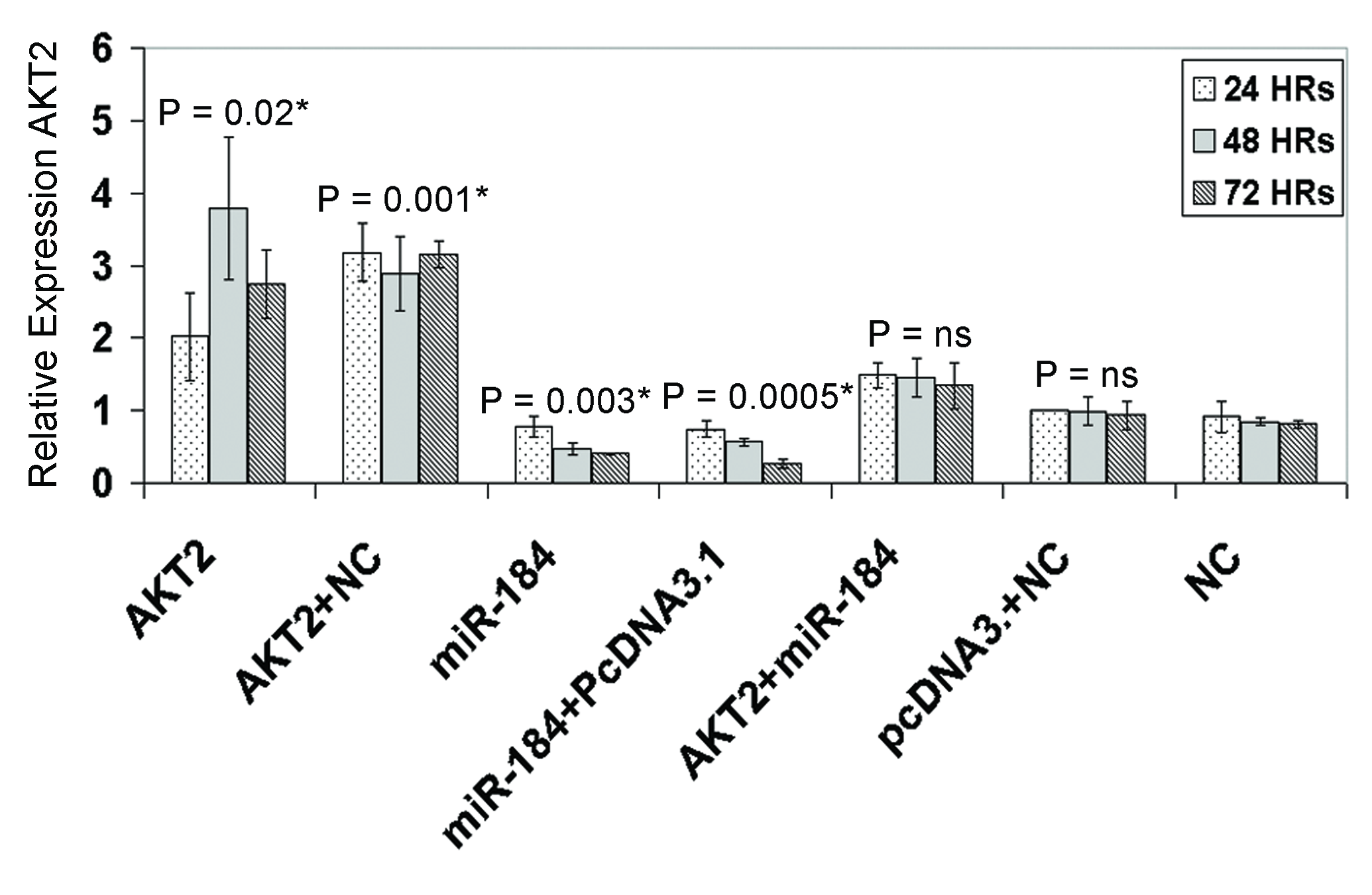

Supplement: Additional file 5 — qRT-PCR assessment of AKT2 mRNA levels at 24, 48 and 72 hours following transfection with different combinations of plasmids and oligonucleotides. In the AKT2 rescue experiment (AKT2 plasmid + miR-184 mimics), AKT2 levels are not significantly different from the negative controls (pDNA3.1 empty vector + negative control oligo or negative control oligo alone) and are intermediate between cells transfected with AKT2 alone (cells having endogenous and ectopic AKT2) and cells transfected with miR-184 mimics alone (inhibited endogenous and no ectopic). All AKT2 mRNA levels are relative to the co-transfection with pcDNA3.1 empty vector and negative control at the 24 hr time point. [file 1476-4598-9-83-S5.TIFF]
